# Radiation Attenuation Performance of Highly Filled Tungsten/TPU Composites via Anchor–Chain Dispersant-Based Interfacial Design

**DOI:** 10.3390/polym18091037

**Published:** 2026-04-24

**Authors:** Seon-Chil Kim

**Affiliations:** Department of Biomedical Engineering, School of Medicine, Keimyung University, 1095 Dalgubeol-daero, Daegu 42601, Republic of Korea; chil@kmu.ac.kr; Tel.: +82-10-4803-7773

**Keywords:** tungsten/TPU composites, anchor–chain dispersant, microvoids, interfacial design, X-ray shielding

## Abstract

Environmentally friendly radiation shielding materials for medical institutions require lightweight characteristics and high flexibility as key performance indicators. One promising approach is the incorporation of lead-free materials that combine high-density shielding fillers with polymer matrices. High filler loading is necessary to maintain shielding performance while preserving the inherent flexibility of the polymer. However, during the mixing of shielding materials with polymers, microvoids may form. Therefore, strategies are required to enhance structural densification of the composite by reducing microvoid formation. This study aims to investigate the effects of interfacial design using an anchor–chain dispersant (APTES: 3-aminopropyltriethoxysilane) on micropore formation, effective density, and X-ray shielding performance in highly filled tungsten/thermoplastic polyurethane (TPU) composites. TPU-based composite shielding sheets containing 75–90 wt% tungsten were fabricated. The dispersant (APTES) can adsorb onto the surface of metal particles and form a stabilization layer. In this study, the observed reduction in particle agglomeration and porosity upon addition of the dispersant suggests that interfacial stabilization was induced. As a result, in the 85 wt% composite sheet, the porosity decreased from 5.89% without the dispersant to 0.56% with the dispersant, leading to an improvement in the densification level and effective density of the sheet. Under the same thickness condition (0.25 mm), the dispersant-containing sheet exhibited a shielding efficiency that was 3–4% *p* higher than that of the sheet without dispersant in the 100–120 kVp range. Meanwhile, as the tungsten content increased, the overall density and shielding efficiency of the sheets also increased. At 90 wt% tungsten loading, the composite demonstrated shielding performance approaching that of conventional lead shielding even at a reduced thickness. These results indicate that interfacial design using an anchor–chain dispersant is an effective processing strategy for improving density uniformity and radiation shielding performance in highly filled tungsten/TPU composite shielding materials by controlling microvoid formation.

## 1. Introduction

The demand for radiation-shielding materials that protect workers and patients has continuously grown in response to the increasing use of X-ray-based imaging, diagnostic examinations, and nondestructive testing in various medical and industrial fields [1,2]. Lead has been extensively used as a shielding material owing to its high density and excellent radiation attenuation properties [3]. However, lead is a toxic-heavy metal; apart from being subjected to increasingly strict environmental regulations, it is difficult to dispose of and recycle. In addition, its high weight and limited processability restrict its use in wearable protective products; hence, developing environmentally friendly shielding materials that can replace lead is an important research objective [4]. Environmentally friendly shielding materials must not only provide shielding performance comparable to that of lead, but also be highly processable and economically feasible. Furthermore, flexibility is an essential requirement when such materials are intended for use in wearable products [5,6].

Composites comprising high-atomic-number metal-particle fillers dispersed within polymer matrices have been proposed as promising alternative shielding materials comparable to lead [7]. Such shielding materials are regarded as environmentally friendly alternatives to conventional ones and offer advantages including low toxicity, high processing flexibility, and superior user comfort when employed in wearable applications [8,9]. Tungsten is widely used as a metal filler owing to its high atomic number, high density, and excellent chemical stability. The excellent X-ray attenuation capability of tungsten has led to its incorporation into polymer matrices to form composite materials that can be processed into various forms, including sheets and films, widely used as eco-friendly shielding materials in recent times [10].

Achieving high shielding performance in composite tungsten–polymer sheets requires significantly high metal-filler loadings. Furthermore, microstructural defects generated during metal-particle/polymer mixing are critical factors that determine shielding performance and composite-material reproducibility [11]. In particular, agglomeration occurs when metal particles (such as tungsten) that are several tens of micrometers or less in size are highly loaded owing to their high surface energies, interparticle attraction, and insufficient interfacial contact with the polymer matrix [12]. Such agglomeration and poor wetting can lead to the entrapment of void spaces around particle clusters during compounding and molding, thereby leading to the formation of microvoids [13].

Microvoids generated during mixing reduce the effective density of the composite shielding material and degrade density uniformity. In addition, microvoids can lead to scattering and non-uniform attenuation along the X-ray path, leading to variations in transmission at the same thickness and a quantitative decline in shielding performance [14,15]. These issues inhibit the development of environmentally friendly composites with sufficient shielding performance. Accordingly, previous studies have attempted to address these issues using processing techniques that improve the densities of composite shielding materials. However, most studies have primarily focused on improving particle dispersion by controlling metal-particle size, which directly influences shielding performance, or via secondary approaches that involve mechanical intervention, such as pressing or degassing [16,17].

Consequently, improvements in shielding performance are often based on limited process indicators, such as particle size and mixing ratio, or mechanical and technical aspects of manufacturing processes, including simple compression molding and casting processes [18,19]. However, calendaring sheet manufacturing, which is a continuous processing method used in practical large-scale production, involves complex compression and shearing conditions that significantly influence how microvoids are formed and collapse. Consequently, tracking microvoid variations by solely examining mechanical processing conditions is a limited strategy [20,21].

While microvoids are generally classified as agglomeration-type, interfacial-type, and bubble-retention-type, they typically appear in complex forms around particle clusters and agglomerated regions when shielding composites are fabricated [22]. Accordingly, in this study microvoid evolution was quantitatively investigated using an anchor–chain dispersant. From a mixing-design perspective, a new processing strategy was introduced for improving shielding performance to reduce microvoid formation by applying an anchor–chain dispersant to a composite shielding sheet composed of tungsten metal particles and thermoplastic polyurethane (TPU).

To improve performance, as the absence of particle-enclosing voids ensures superior density, an interfacial design strategy that enables polyurethane to completely fill the regions that surround the tungsten metal particles in a gap-free manner is required [23,24]. The particle–polymer interface is endowed with certain characteristics, such as wetting behavior, surface oxidation layers (WOx), and surface –OH groups. Polyurethane contains urethane groups (–NH–CO–O–) that participate in polar hydrogen-bonding interactions [25], and the strengths of these interactions must be considered as they affect interfacial stability.

This study aims to reduce microvoids in highly filled W/TPU sheets using an APTES-based anchor–chain dispersant and to establish quantitative correlations among porosity, density, and X-ray attenuation performance. Through this approach, the study seeks to improve the stability of shielding performance in the fabrication process of radiation shielding materials.

The use of an anchor–chain dispersant to suppress particle agglomeration and poor wetting during the tungsten–thermoplastic polyurethane mixing process is expected to reduce microporosity. In addition, this study systematically analyzes the effects of processing variables, such as mixing and forming conditions, on microporosity and density uniformity. Through this approach, causal relationships among porosity, density, and shielding performance are established. Furthermore, this study aims to provide a mechanistic interpretation of microvoid-related issues governing the shielding performance of eco-friendly shielding sheets and to propose a dispersant-based processing strategy to improve radiation shielding performance.

## 2. Materials and Methods

This study analyzes the effect of the internal structure of polymer composites, influenced by the dispersion of metal particles, on the radiation shielding performance in terms of microporosity. Micropores can increase the air volume within the composite, thereby increasing the total volume (V) and reducing the measured bulk density ($\rho_{meas}$) at a constant mass, thereby negatively affecting the shielding performance [26].

In this study, the measured bulk density ($\rho_{meas}$) was calculated from the mass (m) and the geometric volume (V) of the specimen, as shown in Equation (1).

(1) $$\rho_{meas}=\frac{m}{V}.$$

The theoretical density ($\rho_{th}$) of the composite was calculated using the rule of mixtures based on the mass fraction ($\omega_{i}$) and density ($\rho_{i}$) of each component, as shown in Equation (2).

(2) $$\frac{1}{\rho_{th}}=\sum_{i}\frac{\omega_{i}}{\rho_{i}},\;\;\;\;\;\rho_{th}={\left(\sum_{i}\frac{\omega_{i}}{\rho_{i}}\right)}^{-1}.$$

The total porosity (P), including micropores, was defined as shown in Equation (3).

(3) $$P\left(\%\right)=\left(1-\frac{\rho_{meas}}{\rho_{th}}\right)\times 100.$$

Therefore, for compositions under identical conditions, a decrease in porosity results in the measured bulk density ($\rho_{meas}$) approaching the theoretical density ($\rho_{th}$) [27].

The incident radiation intensity ($I_{0}$) entering the shielding material differs from the transmitted radiation intensity (I) passing through the composite. This difference is ascribable to the radiation attenuation effect, which is a function of shielding thickness (χ), and the linear attenuation coefficient (μ), an intrinsic property of the material, according to Equation (4) [28].

(4) $$I=I_{0}e^{-\mu \chi }.$$

The mass attenuation coefficient ($\mu_{m}$) is defined as follows:

(5) $$\mu_{m}=\mu /\rho .$$

Effective density increases with decreasing porosity for composites of identical composition with negligible changes in mass. This increase in effective density results in an increase in the linear attenuation coefficient (μ) according to Equation (6), which consequently leads to improved shielding performance [29].

(6) $$\mu =(\frac{\mu }{\rho })\rho .$$

Tungsten metal particles were used to fabricate radiation-shielding sheets. Tungsten powder (W, 99.9%, 19.3 g/cm^3^, <10 μm, NanGong XinDun Alloys Spraying Co., Ltd., Xintai, China) was crushed for 5 min to control particle size, after which it was dried in an oven at 70–85 °C for 24 h [30]. Prototype sheets were fabricated using TPU as the polymer matrix instead of thermosetting polyurethane. This study aimed to improve particle agglomeration and interfacial characteristics around the particles in highly filled tungsten composites (75–90 wt%) using an anchor–chain dispersant, APTES (3-aminopropyltriethoxysilane, 98%, Duksan CNP, Ansan, Korea), which is an aminosilane belonging to the silane coupling agent family. APTES acts as an anchor that can adsorb and bond to inorganic surfaces through the hydrolysis and condensation of alkoxysilane groups (–Si(OC_2_H_5_)_3_), while the amino group (–NH_2_) promotes interfacial wetting and dispersion stability through polar interactions with TPU [31]. Higher viscosities and inferior processabilities were observed at loadings above 0.5 wt%, whereas values below 0.2 wt% led to particle agglomeration during rolling. Therefore, 0.5 wt% was selected as the optimal content based on the 85 wt% tungsten composition. The anchor–chain dispersant was pre-mixed with the tungsten powder using a pretreatment method to preferentially form a protective adsorption layer on the surface of each tungsten particle during processing.

Dry TPU pellets (PU-95A, Mw. 90,000–450,000, Songwon, Ulsan, Korea) were used as the polymer in this study [32]. TPU melts when heated, thereby enabling the compounding and calendaring to ensure density uniformity and microvoid collapse. In contrast, thermosetting polyurethane (PU) is associated with additional reaction-related variables, including viscosity changes, shrinkage, and heat generation during curing. These factors complicate microvoid tracking and formation mechanisms, which makes it difficult to elucidate the effect of the dispersant [33]. Therefore, TPU was selected as the more suitable matrix material in this study, considering that improving shielding performance through process optimization was an objective of this study.

Tetrahydrofuran (THF, 99.5% purity, Daejung, Siheung, Korea) was used to dissolve the polymer [34]. The evaporation rate was controlled using chloroform (95%, Duksan, Korea) as the co-solvent. All solvents were used without any special pretreatment. TPU was dissolved in THF to a concentration of approximately 10 wt% using a mechanical stirrer, after which tungsten powder was mixed into the prepared casting solution. The particles were uniformly dispersed via ultrasonication (VCX-750, Sonics, Newtown, CT, USA). Impurities were removed by filtration, and the final casting solution was degassed and compression-molded to maintain uniform shielding performance. To remove residual solvents and promote dehydration and solvent evaporation of the shielding sheets, a vacuum drying oven (SH-VDO-70NC, 2020, Sejong, Korea) was used at 60 °C for 12 h, thereby minimizing the residual solvent.

**Figure 1:**
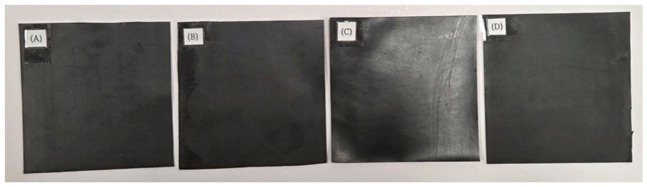
Photographic images of sheets fabricated by calendaring at tungsten contents of (**A**) 75, (**B**) 80, (**C**) 85, and (**D**) 90 wt%.

Shielding sheets were calendared to a target thickness of 0.25 mm. Figure 1 shows that sheets prepared using various tungsten loadings are virtually indistinguishable by visual inspection. Two types of 85-wt–containing tungsten sheet (with and without dispersant) were prepared to investigate the effect of the anchor–chain dispersant used in this study. In addition, four types of sheets with tungsten contents of 75, 80, 85, and 90 wt% were fabricated to investigate how internal density and shielding performance are affected by the metal-particle content. The tungsten content was selected based on a high loading criterion, considering two main factors—sheet stability and flexibility. After the calendaring densification process, the sheets maintained flexibility up to a maximum tungsten content of 90 wt%. In addition, a minimum tungsten content of 75 wt% was required to ensure stability during the THF-based solution processing. This composition range allowed for a systematic evaluation of the dispersant effect across different loading levels and was therefore selected for this study. The calendared sheets were fabricated with dimensions of 1.2 m × 1.2 m, and the sheets used for the experiments were cut into specimens of 0.3 m × 0.3 m. These sheets containing the anchor–chain dispersant were labeled W-TPU 75, W-TPU 80, W-TPU 85, and W-TPU 90, respectively. This approach facilitated determining the optimal dispersant conditions for the composite formulation.

**Figure 2:**
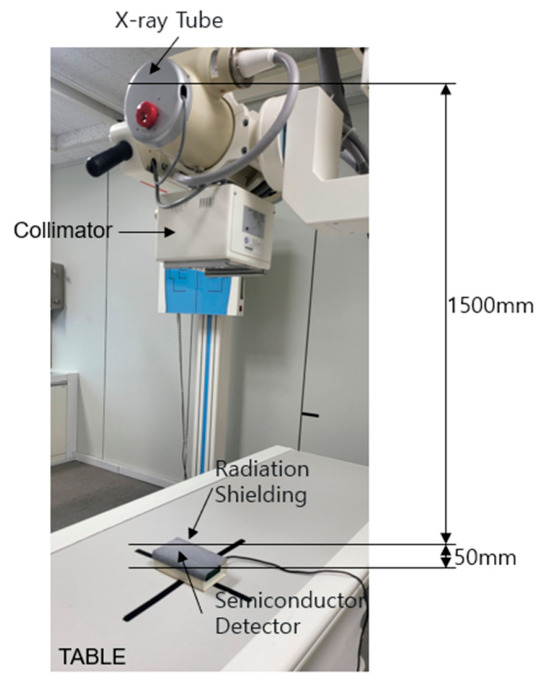
Photographic image of the equipment used to evaluate the radiation-shielding performance of the various shielding sheets fabricated in this study.

The particle dispersion within each fabricated shielding sheet was observed by preparing a thin cross-sectional specimen and examining it via field-emission scanning electron microscopy (FE-SEM, Hitachi S-4800, Hitachi High-Tech, Tokyo, Japan) [35]. The experimental configuration used to evaluate radiation-shielding performance is shown in Figure 2, and it follows the KS A 4025 Korean Industrial Standard (Method of test for lead equivalence of X-ray protective materials; 1990, confirmed in 2009) [36]. The distance between the X-ray generator and the shielding sheet was set to 1.5 m, and that between the shielding sheet and the detector was maintained at 0.05 m to minimize backscattered radiation. The X-ray generator used was Toshiba E7239 (150 kV–500 mA, 1999, Toshiba Medical Systems, Otawara, Tochigi, Japan), which is commonly used for diagnostic purposes in medical institutions. Each experiment was repeated 10 times, and the average value was used for the analysis. The radiation dose was measured using a DOSIMAX Plus A semiconductor detector (Scanditronix, Wellhofer, Schwarzenbruck, Germany) after proper inspection and calibration [37].

The shielding performance of each sheet was evaluated in terms of its radiation protection efficiency (RPE) under various conditions according to Equation (7) [38].

(7) $$RPE=(1-\frac{e}{e_{0}})\times 100,$$

where e is the radiation dose (μR) measured when the shielding sheet is placed between the X-ray beam and detector, and $e_{0}$ is the radiation dose (μR) measured in the absence of the shielding sheet. A standard lead sheet was tested under the same conditions as a reference.

## 3. Results

Bulk densities (based on the tungsten ratio in the polymer matrix) were calculated to compare the effective densities of sheets fabricated with and without the dispersant. The apparent densities used to determine porosities are also presented according to the presence or absence of the dispersant. As shown in Table 1, the theoretical density of the fabricated sheet containing tungsten (W-TPU 85 wt%) calculated using the rule of mixtures was 11.652 g/cm^3^. The reported density value corresponds to this calculated value, whereas the porosity was determined based on the measured density obtained from the specimen mass and geometric volume. With the application of the dispersant, the porosity decreased from 8.59% to 0.56%. Accordingly, the total porosity was calculated using the measured bulk density and theoretical density, and the error was presented as the standard deviation obtained from repeated measurements.

Table 2 shows that density increases with increasing tungsten content at the same sheet thickness, which reveals that the dispersant within the polymer matrix becomes more effective with increasing tungsten-particle content. A tungsten content of 75 wt% corresponded to a density of 10.402 g/cm^3^, while a value of 12.801 g/cm^3^ was recorded at 90 wt%.

**Figure 3:**
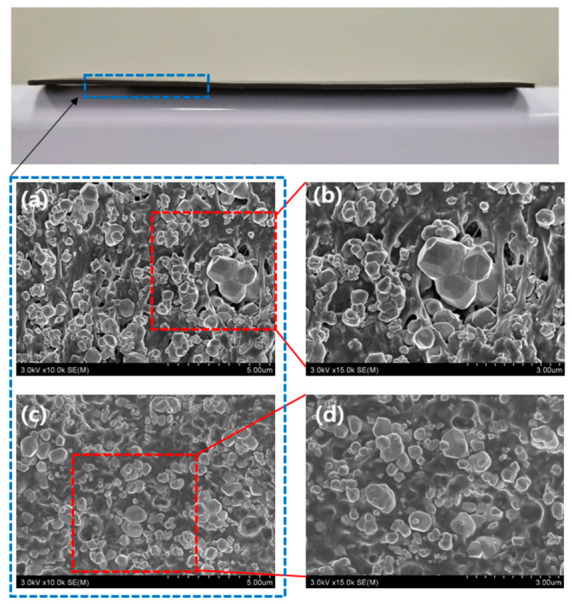
Cross-sectional SEM images based on the presence or absence of the anchor–chain dispersant: (**a**) without the dispersant, (**b**) a magnified view of (**a**), (**c**) with the dispersant, and (**d**) a magnified view of (**c**).

The SEM images of the W-TPU 85 samples displayed in Figure 3 were used to analyze interfacial metal-particle/polymer-matrix structures in terms of the presence or absence of the anchor–chain dispersant. Ideally, the polymer should uniformly surround each tungsten particle; satisfyingly, such a well-dispersed structure is shown in Figure 3b. In contrast, the sheet fabricated without the dispersant exhibited agglomerated tungsten particles and an aggregated polymer phase (Figure 3a), indicative of poor metal-particle dispersion in the polymer matrix.

**Figure 4:**
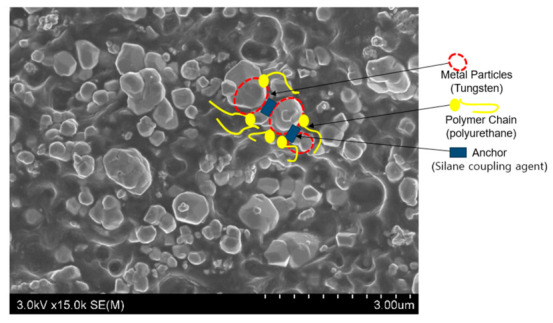
SEM image depicting the internal structure of a W-TPU 85 sample containing the anchor–chain dispersant.

Figure 4 reveals that the W-TPU 85 sheet with the anchor–chain dispersant exhibits a structure in which the dispersant is stably anchored to the surfaces of the particles, enabling the polymer chains to effectively bind to the metal particles. The dispersant is intended to stabilize the metal-particle/polymer-matrix interface and reduce microvoid formation, resulting in a dense polymer microstructure. Consequently, Figure 4 shows that the metal particles are uniformly and stably encapsulated by polymer chains.

The particle dispersion behavior based on the presence of the dispersant was analyzed through image analysis. The agglomerate area fraction was compared using the image processing software Image J (version 1.53, NIH, Bethesda, MD, USA). The same algorithm was used to perform background correction and connected component analysis to separate agglomerates according to the size criteria, and the area fractions were compared under identical conditions, yielding the results shown in Table 3. The images obtained with the dispersant showed fewer agglomerates. In contrast, the overall tungsten phase fraction appeared higher in the samples without the dispersant, indicating that the bright tungsten phases were more extensively segmented in those images.

The fabricated sheets show slight dispersant-dependent shielding efficiencies. A higher effective density and fewer microvoids within the sheet effectively led to more shielding material along the X-ray transmission path at the same thickness. These effects mitigated any decrease in RPE, even under conditions where shielding performance typically deteriorates with increasing X-ray energy. Table 4 reveals that the dispersant-containing W-TPU 85 sheet exhibited RPE values that are three to four percentage points higher than those of the sheet devoid of dispersant in the 100–120 kVp range, confirming that microvoid control advantageously maintains shielding performance under high-energy conditions.

**Figure 5:**
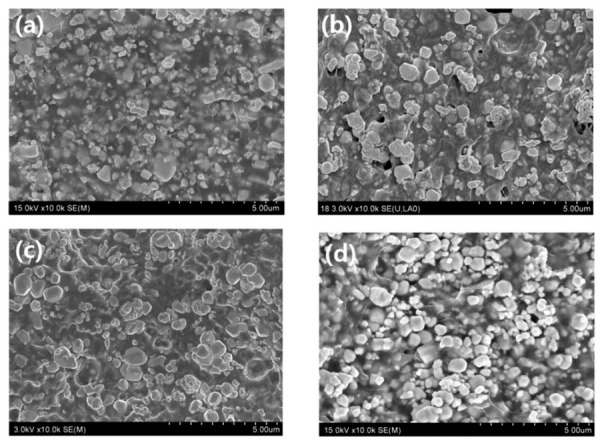
SEM images showing internal particle dispersions at tungsten contents of (**a**) 75, (**b**) 80, (**c**) 85, and (**d**) 90 wt%.

Figure 5 reveals that the inter-filler space decreases with increasing tungsten content, while the amount of the continuous polymer matrix phase that fills this space and the average interparticle distance decrease, resulting in increased particle–particle contact. A lower relative proportion of the polymer-matrix was observed in the 75–80 wt% range, leading to more particle clustering and locally concentrated regions exhibiting partial microstructures. The TPU matrix was relatively continuously distributed in the 85–90 wt% range, with tungsten particles transitioning into a relatively uniformly dispersed particle-dominated structure throughout the entire region. In addition, the voids or gaps observed around some particles suggest the possible presence of microvoids formed by incomplete wetting and air entrapment at high filler loadings.

Table 5 presents X-ray shielding performance data for sheets with tungsten contents of 75–90 wt% at a thickness of 0.25 mm, expressed in terms of the RPE. All sheets exhibited decreasing RPEs with increasing tube voltage (kVp), consistent with the general observation that attenuation decreases with increasing incident X-ray energy at constant thickness. In contrast, the RPE increased with increasing tungsten content at the same tube voltage, confirming that a high-atomic-number filler loading contributes directly to shielding performance. The RPE increased from 85% for W-TPU 75 to 98% for W-TPU 90 (ΔRPE = 13 percentage points) at 40 kVp, while it increased from 75% to 89% (ΔRPE = 14 percentage points) at 120 kVp. Notably, the RPE ranged between 86% and 95% across all energies at a tungsten content of 85 wt% or higher, confirming that the highly filled region exhibited significantly improved shielding performance. These results are attributable to the higher effective density and higher probability of X-ray interactions at high tungsten contents, leading to lower X-ray transmissions. Furthermore, a decrease in the performance gap relative to that of lead was observed with increasing tungsten content. In particular, high shielding performance was achieved at a tungsten content of 90 wt%, even for a 0.25 mm thin sheet, suggesting its potential as an alternative to conventional lead shielding materials.

## 4. Discussion

**Figure 6:**
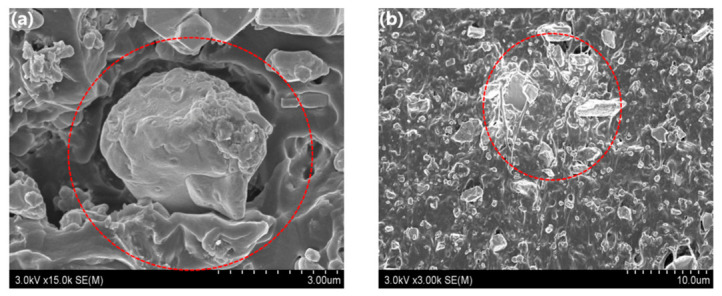
SEM images showing poorly dispersed particle distributions ascribable to (**a**) air trapped at the interface, and (**b**) polymer aggregation in the absence of metal particles.

A dispersant was used to enhance shielding-filler dispersion and improve the shielding performance of radiation-shielding composites. The dispersant acts as an agent that removes trapped air pockets at interfaces in a similar manner to conventional vacuum degassing, thereby preventing metal-particle agglomeration [39,40,41]. Therefore, in this study, a polymeric dispersant with an anchor–chain structure was used. This dispersant is strongly adsorbed onto the surfaces of the tungsten particles, thereby preventing particle agglomeration and improving compatibility with the polymer matrix, which helps reduce void formation and particle aggregation [42]; this effect also increases the internal density of the material. Consequently, the dispersant is expected to simultaneously reduce microvoids and interfacial gaps. Figure 6a shows that the metal particles tend to adhere to each other owing to their high surface energies, with air pockets trapped around fine agglomerates. Such structural defects ultimately lead to shielding-performance deterioration (Figure 6b). Uniform metal-particle dispersion can be prevented by polymer aggregation.

The dispersant forms protective layers around the metal particles to prevent their re-agglomeration, thereby improving the shielding performance; it facilitates control over initial microvoid formation and particle dispersion through judicious interfacial design. Weak anchor interactions or insufficient chain lengths may result in inadequately maintained interparticle distances, leading to particle re-agglomeration [43]; consequently, an appropriate dispersant must be used. Microvoid control is determined more by polymer chain mobility and interfacial bonding than by process-related factors such as compression or degassing [44]. As demonstrated in this study, promoting internal dispersion via microvoid control and particle rearrangement addresses these issues, thereby improving shielding performance. The formation of microvoids in highly filled shielding sheets can be prevented by forming a protective (adsorption) layer around each metal particle to prevent particle re-agglomeration, while simultaneously minimizing interfacial gaps by improving interfacial wettability [45,46]. The anchor–chain dispersant used in this study provides an effective means of satisfying these requirements.

The reduction in microvoids through the application of the dispersant is not limited to microstructural improvement but is also directly related to density and radiation attenuation characteristics. In addition, quantitative SEM analysis confirmed that the incorporation of the dispersant led to a decrease in the agglomerate area fraction and enhanced the particle dispersion stability. This can be attributed to the reduced probability of local low-density regions and interfacial void formation, which helps improve the uniformity of in-plane attenuation performance.

Among the shielding sheet fabrication processes proposed herein, the application of the dispersant is intended to reduce internal micropores in the sheet, ultimately improving the shielding performance. Protective garments for medical personnel must ensure mobility through flexibility and lightweight properties, and the proposed approach is expected to positively contribute to these functional requirements. The proposed method is expected to provide a more quantitative and reproducible standard approach to reduce porosity and increase density compared with the previously reported methods. The microvoid changes were mainly evaluated by comparing cross-sectional FE-SEM images, which is an inherent limitation of this study. Accordingly, a diverse array of quantitative characterization methods is required to further validate our conclusions. Further studies involving various types of polymer and metal particles are necessary, while microstructural changes need to be tracked more quantitatively. Moreover, three-dimensional porosity measurements using micro-computed tomography (micro-CT) are expected to enable quantitative analyses of particle dispersions. In addition, further validation, including quantitative analysis of the residual solvent during the casting process of sheet fabrication, is necessary.

Previously reported lead-free polymer-based X-ray shielding composites mainly utilize fillers such as W, Bi_2_O_3_, BaSO_4_, and Sn/Sb systems, and their shielding performance (μ, HVL, and Pb-equivalent) varies depending on the filler loading and processing methods (casting, extrusion, or pressing) [47,48]. This study demonstrated the role of the dispersant in highly filled tungsten metal-particle composites. The superiority of the proposed approach was confirmed through various objective indicators, such as the reduction in microvoids, recovery of measured bulk density, and improvements in porosity and shielding performance, highlighting the importance of microvoid reduction.

While the development of environmentally friendly radiation-shielding materials initially aimed to deliver shielding performance comparable to that of lead, achieving superior material properties that transcend those of lead is the ultimate goal. Metal-particle dispersion is a critical factor that significantly influences shielding performance and serves as a key performance indicator of a polymer-based shielding composite [49,50]. Therefore, an optimized mixing process that ensures structural stability is required to deliver a uniform dispersion and stable particle arrangement [51]. While discovering new metal fillers is important, further research into metal-particle/polymer-matrix interfacial compatibility is also necessary.

## 5. Conclusions

This study demonstrated that the anchor–chain dispersant (APTES: 3-aminopropyltriethoxysilane) effectively reduced micropores in highly filled tungsten/TPU composite shielding sheets by mitigating particle agglomeration and interfacial defects, thereby improving sheet densification and effective shielding performance. In particular, porosity decreased from 8.59% to 0.56% when the dispersant was included in the 85 wt% tungsten-particle-containing sheet. Furthermore, the dispersant-containing sheets exhibited shielding efficiencies that were 3–4 percentage points higher than those of the dispersant-devoid sheets in the 100–120 kVp range, confirming the effectiveness of interfacial-design-based dispersion control. In addition, shielding performance was observed to generally improve with increasing tungsten content. These results reveal that optimizing the performance of highly filled metal/polymer composites not only requires consideration of the filler content but also dispersant-based interfacial control as a key design feature.

## Figures and Tables

**Table 1:** Properties of W-TPU 85 sheets fabricated with or without the anchor–chain dispersant.

	Weight (kg/m^2^)	Thickness (mm)	$\rho_{meas}$ (g/cm^3^)	$\rho_{th}$ (g/cm^3^)	Porosity (%)
With dispersant	2.900 ± 0.035	0.250 ± 0.012	11.587 ± 0.012	11.652	0.56 ± 0.10
Without dispersant	2.900 ± 0.020	0.250 ± 0.021	10.651 ± 0.012	11.652	8.59 ± 0.10

**Table 2:** Properties of four types of fabricated sheets with different tungsten contents.

	Weight (kg/m^2^)	Thickness (mm)	Density (g/cm^3^)
W-TPU 75	2.600 ± 0.020	0.250 ± 0.007	10.402 ± 0.010
W-TPU 80	2.700 ± 0.012	0.250 ± 0.004	10.804 ± 0.021
W-TPU 85	2.900 ± 0.025	0.250 ± 0.012	11.652 ± 0.012
W-TPU 90	3.200 ± 0.010	0.250 ± 0.013	12.801 ± 0.012

**Table 3:** Comparison of agglomerate area fraction through image analysis according to the presence of the anchor–chain dispersant.

Anchor–Chain Dispersant	Area Fraction of Particle Clusters (%)	Area Fraction of Agglomerates (%)
Contains	18.82	1.68
Not included	22.41	3.28

**Table 4:** Shielding properties of fabricated W-TPU 85 sheets with and without the anchor–chain dispersant.

X-Ray Energy (kVp) Tube Voltage	RPE (%) (Thickness: 0.25 mm)	
	With Dispersant	Without Dispersant
40	95	95
60	92	91
80	90	89
100	88	85
120	86	82

**Table 5:** Shielding properties according to tungsten content.

X-Ray Energy (kVp) Tube Voltage	RPE (%) (Thickness: 0.25 mm)				
	W-TPU 75	W-TPU 80	W-TPU 85	W-TPU 90	W-TPU 95
40	85	90	95	95	100
60	75	88	92	92	98
80	82	85	90	90	95
100	79	84	88	88	94
120	75	80	86	86	91

## Data Availability

All data generated during this study are included in this manuscript.

## Abbreviations

The following abbreviations are used in this manuscript:

TPU	Thermoplastic polyurethane
PU	Polyurethane
THF	Tetrahydrofuran
FE-SEM	Field-emission scanning electron microscopy
RPE	Radiation protection efficiency
